# Nutritional Properties of Wild Edible Plants with Traditional Use in the Catalan Linguistic Area: A First Step for Their Relevance in Food Security

**DOI:** 10.3390/foods13172785

**Published:** 2024-09-01

**Authors:** Mar Casas, Joan Vallès, Airy Gras

**Affiliations:** 1 Laboratori de Botànica—Unitat Associada CSIC, Facultat de Farmàcia i Ciències de l’Alimentació—Institut de la Biodiversitat IRBio, Universitat de Barcelona, 08028 Barcelona, Catalonia, Spain; casasmadridmar@gmail.com (M.C.); joanvalles@ub.edu (J.V.); 2 Secció de Ciències Biològiques, Institut d’Estudis Catalans, 08001 Barcelona, Catalonia, Spain

**Keywords:** Catalan linguistic area, ethnobotany, dietetics, nutrition, traditional knowledge, wild edible plants, wild food plants

## Abstract

Wild food plants (WFPs) are crucial for the subsistence of many human populations. While there are at least 7000 edible plant species in the world, only approximately 420 are considered food crops. WFPs are often studied from the phytochemical and pharmacological point of view, because they include available food components with nutraceutical value. The present study aims to highlight the nutritional value of WFPs traditionally used in the Catalan linguistic area, providing detailed insights and discussing the significance of these properties. Information about the nutritional properties of 93 taxa, coming from ethnobotanical prospection, has been collected through an extensive bibliographic research. The results reveal that WFPs are rich in nutrients, especially micronutrients. Furthermore, in selected species, those for which nutritional information and a cultivated homologue are available, the nutrient content in wild taxa exceeds than of phylogenetically related crop plants with similar use. Traditional wild plant preparation forms for food and the nutritional value of a menu constituted by wild food plants are presented. This research represents a preliminary step toward selecting certain taxa that could be developed into new small- or large-scale crops or sustainably harvested in the wild, contributing to food security.

## 1. Introduction

There are at least 7039 edible plant species in the world, but only 417 are considered food crops and, according to the Food and Agriculture Organisation of the United Nations [1], just 15 crop plants contribute to 90% of humanity’s intake, and more than four billion people rely on rice, maize and wheat [2]. By 2050, it is anticipated that the global population will have increased tenfold since 1800 and there is a need to find new, sustainable ways to feed the global population [2].

Wild food plants (WFPs) play an important role in the subsistence of many human populations [3,4,5] and also are an important part of traditional food systems, having a very important cultural value for many groups of people [3].

In order to gain a clear idea, wild food plants is the concept used to refer to all plants with parts, whether roots, leaves, stems, fruits or others, that can be consumed by the general population. These plants often have obvious nutritional properties and are, organoleptically speaking, pleasant. Noteworthy, wild edible plants include available food composition parameters with, in some cases, nutraceutical values. This is the reason why they are often studied from the phytochemical and pharmacological points of view [6].

However, some studies are focused on the growing loss of traditional knowledge on wild food plants [6,7]. The population that is currently known to be interested in harvesting these plants is basically elderly people. These are the experts and the ones in charge of passing on all their knowledge to future generations, but acculturation, frequently affecting industrialized areas, applies in this case and makes this vertical transmission of traditional plant knowledge weak, and often weaker [8].

Ethnobotany, in the interface of the social and natural sciences [9], is the science of studying the cultural use of plant materials. This discipline deals with all kinds of traditional knowledge about the use and processing of plants by human populations, including the WFPs.

The use of WFPs in the Catalan linguistic area (CLA) is closely related to the traditional popular culture of the different locations. In fact, this current use comes from a very ancient tradition, rooted and at least documented from mediaeval times, with works that have become worldwide classical in the culinary literature, such as the ‘Llibre de Sent Soví’, a compilation of 13th–14th-century recipes, originally written in Catalan and then translated to other languages, such as Spanish and French [10]. From this early experience, the Catalan cuisine is the reflection of a society and of the Mediterranean land where it is located [11]. In a previous work performed in the CLA, a total of 291 taxa were quoted as WFPs [8]. This corpus constitutes a significant part of the edible resources in the territory considered, sometimes underestimated and underutilized.

Although there is an important amount of literature on WFPs, most studies are focused on the pharmacological properties of those plants [12,13,14], but not so much on their nutritional properties, even though they are often very related. Some WFP species contain more micronutrients than other cultivated ones, the perfect pretext to promote their production, distribution and consumption and to highlight that another agri-food model is as possible as essential [15].

The present work aims at highlighting the nutritional value of the corpus of WFPs in CLA [8], with the aim of deepening into the details and discussing the importance of the properties and the use that may be given to them. The specific aim of this investigation was to study wild food plants and assess their nutritional properties, through an extensive literature survey on a set of plants built from our ethnobotanical data on WFPs in the CLA. In addition, this work aimed to recover and preserve the heritage of the traditional knowledge about these wild species, indicating—and vindicating—them as potentially very important in feeding humanity.

## 2. Materials and Methods

### 2.1. Study Area

**Figure 1:**
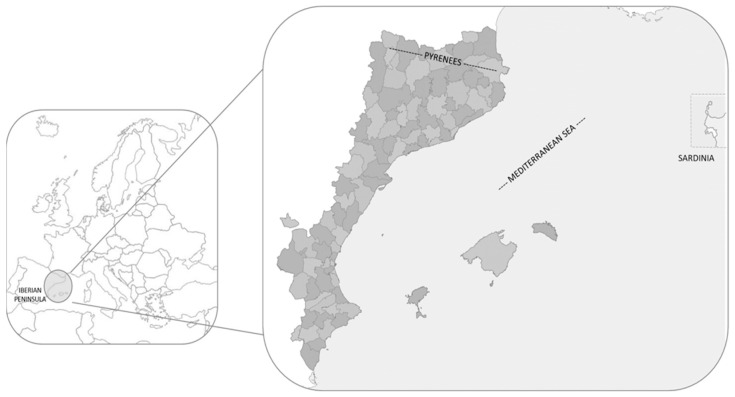
Catalan linguistic area and its location in Europe.

This paper is focused on the CLA, a territory that has been well studied at geographical, cultural, linguistic, botanical and gastronomic levels [11,16,17,18,19,20,21,22] (Figure 1). The region, also referred to as Catalan-speaking or Catalan-language territories, is the concept used to refer to the north eastern portion of the Iberian Peninsula, a north Pyrenean part, the Balearic Islands and the city of l’Alguer in Sardinia.

It covers approximately 70,000 km^2^ from the level of the Mediterranean Sea to the Pyrenees at 3145 m a.s.l. [20]. This provides great diversity in terms of flora and fauna and allows the presence of a large number and diversity of species within the same territory. The approximate number of plant taxa (including species and subspecies) in these territories is 4300 autochthonous plus 1200 allochthonous [23].

### 2.2. Databasing and Data Selection

The list of 153 taxa that have been quoted in a meta-analytical paper on wild food plants (WFPs) in the CLA [8]. This work was performed to identify the WFPs used in the CLA, and the ones studied in the present work are those with three or more use reports, according to one factor among reliability criteria [24,25]. For more details about how the ethnobotanical data were collected, check Gras et al. [8].

### 2.3. Nutritional Databasing and Data Selection

Information on the use and nutritional properties for each taxon has been collected through an extensive literature search, aiming at obtaining all the published nutritional data for the set of taxa object of this study. The target of the dietetic analyses to be consulted has been macronutrients (available carbohydrates, proteins, and lipids), micronutrients, energy, and fiber. The works used comprised phytochemical research where the content of plants is analyzed, books with the same content (e.g., with tables of food composition) and occasionally recipe books with a dietary perspective [26,27,28,29,30,31,32]. In most cases, the results obtained in these analyses were on the total fresh content of the plant (including water). In others, the ratio was based on the total content of the dry plant (without water). For dry matter data, and in order for all the data collected to be equivalent, the fresh content of the plant had to be calculated through the water content of the same, which was collected in the research consulted [33].

Regarding the micronutrients, the investigation focuses around the most representative and takes into account the nutritional role that they play in the human organism.

## 3. Results and Discussion

From all 291 taxa recorded in Gras et al. [8], only those with more than three use reports were selected following the reliability criteria [24,25]. That left 52.6% of species (153 taxa) to analyze. Nutritional information on 93 species was found (60.8% of all the taxa analyzed). Of these 93, 80 (86% of all the taxa) have taxon-specific information, that is unique data for each taxon exactly coincidental with that quoted in Gras et al. [8]. The other 13 (14%) have extrapolated information from another species of the same genus (12 taxa) and general information for the genus (one taxon). It is noteworthy, then, that there are not enough data for 60 taxa of relevance.

Therefore, all this information has been put together in databases of their own (Supplementary Materials, Tables S1–S6), for nutritional information about the species that constitute our source of analysis, and for nutritional data from species of the same genus as some plants quoted in Gras et al. [8] that lack specific nutritional information.

### 3.1. Nutritional Properties and Requirements

Plants vary in their appearance and structure, so it is obvious they also differ in their composition. At a first glance, there are some plants that stand out from the rest for certain nutrients.

The species with the highest energy presence, in terms of calories, is *Pinus pinea* L., with a contribution of 678 kcal per 100 g of seed (Table S1), which is the part of the plant that is consumed, pine nut (in Catalan ‘pinyó’). In fact, it is the same plant that stands out for its lipidic content. It is logical, then, this is the plant that provides more energy, since fats are the elements that provide more calories per gram (9 kcal/g) [34].

When analyzing carbohydrates and their content in different plants, *Arundo donax* L., belonging to the family of the Poaceae, appears as the most dominant. It provides 82.6 g of carbohydrates for every 100 g of shoot (Table S1), one of the plants parts used as a condiment. *Arundo donax* is a plant that is being widely used as an alternative to other sources of wood fiber in the paper industry, due to its high content of carbohydrate constituents: cellulose, hemicellulose and uronic acids [35]. Despite this, its food use is still hard to find [36].

Since the species with the highest proportion of sugars is used as a condiment, but not ingested, the second most relevant plant becomes the target to analyze. *Laurus nobilis* L., despite containing an amount of 75 g of carbohydrates per 100 g of fresh leaves, is not usually directly consumed as a food product (but just its active principles) and therefore cannot have much effect on the body. The third species is *Origanum vulgare* L. (69 g of carbohydrates per 100 g of fresh leaves). Although it is also used as a condiment, it is ingested in pizzas, pastas, breads, etc., so it can have repercussions in the human body.

Finally, in reference to the macronutrients, the predominant species in terms of protein content is *Chamaerops humilis* L., providing 30.3 g of protein per 100 g of fruit (Table S1). This high content in protein suggests that *Chamaerops humilis* fruits are a good source of basic macronutrients. The amount of protein in *Vicia sativa* L. (28.3 g of protein per 100 g of dry seeds) is also remarkable. This species belongs to the Fabaceae family, which includes other crop species like *Glycine max* L. or *Vicia faba* L. The protein content of *Vicia sativa* is not far from the 35.9 g of protein per 100 g of dry seeds of *Glycine max* or 26.1 g in *Vicia faba* seeds.

Reference dietary intakes include a set of standardized reference values for intakes of the adult and healthy population. These values include the recommended dietary allowance (RDA), adequate intake (AI) and tolerable upper intake (UL). Table 1 describes these values for the micronutrients that are most represented in this paper and for which there are clear nutritional recommendations, their respective dominant taxa, its content and its relation to dietary reference intakes. [37]. Macronutrient recommendations will not be taken into account in this table, since their recommendations vary greatly depending on individual aspects (weight, sex, age, etc.).

This discussion is based on plant quantities of 100 g. Many of these plants are not consumed in the form of rations, but as a condiment or in other types of preparations that require small amounts of the plants. Therefore, it should be understood that the reference dietary intake percentages are indicative. In addition, in some micronutrients, the requirements are very low and are covered with very little food intake, but they are far from approaching toxic amounts. This is the case with vitamin B3 or niacin, manganese, copper or zinc.

*Satureja hortensis* L. is the species that stands out the most in terms of nutritional content, with the highest content of dietary fiber (45.7 g) and magnesium (377 mg). Dietetic fiber is a non-digestible carbohydrate chain with many proven beneficial properties. Among them, fiber is known to increase satiety, improve the glycemic index of food intakes, and reduce the risk of developing DM2 in the long term [42]. Magnesium is a mineral involved in more than 300 enzymatic reactions regarding protein synthesis, muscular contraction, nervous function, glucose control and many more [43]. Not surprisingly, this plant belongs to the Lamiaceae family, known and used traditionally for its wide range of chemical compounds with different biological activities. Correspondingly, and because of their essential oils, these types of plants have been historically used for their ability to act as flavoring agents in cuisine [44].

On the other hand, *Allium roseum* L. dry bulbs, are also distinguished for their high content of potassium (1530 mg), essential in the cellular function maintenance, muscular contraction and neuronal conduction [45]; and vitamin B1 or thiamine (239 μg). Vitamin C is one of the most important reducing agents in the body, it gives up an electron to an organic substrate in order to reduce oxidation. It is found in greater concentration in the dry bulbs of *Allium roseum* L. (1523 mg). It should be noted that this vitamin is degraded with heat and, therefore, it is important to consume these leaves raw to obtain the maximum contribution of the nutrient [46].

Following up, sodium is highly present in fresh flowers of *Capparis spinosa* L. (2960 mg). It is involved in kidney function, the hormones of fluid balance and, therefore, in the body’s general homeostasis. This way, sodium can cause effects on blood pressure. An excess of sodium has adverse and direct consequences in increasing the risk of hypertension [47].

Calcium, also highly present in *Urtica urens* L. (4421 mg), is involved in bone health and its mineral density. Its intake is increased when consumed along with dairy products, so the combination of the plant with these products becomes interesting. Vitamin D becomes an essential compound to ensure absorption and maintain normocalcemia [48]. If adequate levels of calcium are not achieved, this begins to be released from the bones and teeth, producing osteopenia at first and osteoporosis in a prolonged manner [49]. This taxon also stands out for its content in manganese (10,400 μg). Manganese is an essential metal for the human body. It intervenes in immune function, blood sugar regulation, coagulation and defense against reactive oxygen species. This metal incorporates its properties into the functioning of the body through metalloproteins, which makes manganese one of the most common metals in tissues [50].

*Salvia officinalis* L. leaves also show dominance in iron content (84 mg). Iron plays an important role in many metabolic processes, like oxygen transportation, DNA synthesis and electron transportation.

*Chondrilla juncea* L. stands out for its concentration in cobalt (430 μg), which plays a very important role in the constitution of vitamin B12, essential in DNA synthesis and energy production [51].

Phosphorus is found in large proportions in the fresh seeds of *Pinus pinea* (650 mg). It is an essential component in the structure of bones, cells and genetic material [52].

*Corylus avellana* L. fresh seeds possess an elevated zinc (2500 μg). Zinc constitutes an indispensable element in many biochemical functions. Its deficit, then, disturbs several organ systems, such as the epidermal, gastrointestinal, central nervous, immunological, skeletal and reproductive systems [53].

Vitamin A is found in greater ratio in *Anethum graveolens* L. fresh leaves (7720 IU). This vitamin is part of a group of essential micronutrients essential for the human body, so it needs to be collected from diet. It is indispensable in maintaining the integrity and functionality of the epithelium. It also intervenes in the visual function, especially in low light conditions; also in the health of the immune system and in the correct growth, development and reproduction. Vitamin A deficiency increases the risk of infection, which increases the demand of vitamin A at the same time. Children are a population at high risk for this vicious cycle, which explains why vitamin A deficiency is such an important cause of infant mortality [54].

*Coriandrum sativum* L. has a distinguished content of vitamin B1 (239 μg) and vitamin B3 (2130 μg). Thiamine (vitamin B1) constitutes an indispensable coenzyme for carbohydrate, lipids and protein metabolism. Its deficiency causes mitochondrial dysfunction and lactate and pyruvate accumulation, which can lead to neurologic and cardiovascular complication [55]. Riboflavin (vitamin B2) is a water-soluble vitamin known for its antioxidant, anti-aging, anti-inflammatory and antinociceptive properties (reversal of pain feeling). It is found in greater concentration in the leaves of *Plantago major* L. (300 μg) [56].

*Carum carvi* L. fresh seeds stand out for their vitamin B6 content (360 μg). This vitamin plays an essential role in the maintenance of carbohydrate metabolism, DNA synthesis, the generation of antioxidants and epigenetic regulation. Deficiencies in this nutrient can result in affectations on development, cognitive function or blood production [57].

Vitamin B9 is found in greater amount in *Allium ampeloprasum* (327 μg). Vitamin B9 is involved in the synthesis, stability, integrity and repair of DNA. Deficiency in folate (vitamin B9) results in hypersegmented neutrophils, which lead to megaloblastic anemia [58,59].

*Artemisa absinthum* L. stands out for its content in vitamin E (27,405 mg). This, provides an important antioxidant activity in the human organism, although it needs to be obtained exclusively from the diet [60]. This plant is largely cultivated, but its wild populations could constitute a good complementary source.

The fresh leaves of *Rorippa nasturtium-aquaticum* L. stand out for their vitamin K content. Coagulation factors, osteocalcin and anticalcifying proteins functions directly depend on this vitamin [61].

Some species contain significant amounts of other micronutrients for which there are no clear reference dietary intakes, but they have been proved to be essential for the proper functioning of the body.

The ingestion of carotenoids provides benefits against many health problems, including neurological disorders, heart complications or infections, among others [62]. These are found predominantly in *Salvia officinalis* (3480 μg), used as a condiment that is ingested, and *Beta vulgaris* L. subsp. *maritima* (L.) Arcang. (14.3 mg), used in salads. Flavonoids are associated with antioxidative activity, free-radical scavenging capacity, coronary heart disease prevention and anti-cancer activity [63]. *Crataegus monogyna* Jacq. fruits have appeared to be outstanding for this nutrient (110 mg CAE).

*Crithmum maritimum* L., usually consumed preserved in vinegar as an appetizer or in salads, is the plant that appears to provide more phenolic compounds (1260 mg GAE). Phenolic acids have been displayed to have their own antioxidant potential within free-radical oxidation reactions. They have also been found to promote the anti-inflammation capacity of human bodies [64].

Nitrates and their anions have been proved to contribute to the health of the gastrointestinal tract, cardiovascular system and inflammation [65]. They appear most present in leaves of *Eruca vesicaria* L. (257 mg), used as a salad component.

### 3.2. Plants Involved in Traditional Food Preparations

Many of the plants mentioned in this paper are involved in traditional preparations within the gastronomic culture of our country. Table 2 lists some preparations with plants that have been named so far and for which nutritional information has been recorded [8].

With the aim of revaluing these plants, with possibly more importance within the current agri-food framework, it is considered to design a menu in which each dish includes the plants for which nutritional information has been found. The recipes have been retrieved from [66]. The nutritional value of the entire menu can be found in Table 3.

1. Starter: *Portulaca oleracea* salad;
2. Main: rice with *Silene vulgaris* and other vegetables;
3. Dessert: *Arbutus unedo* pie.

The menu is, in general, more complete in terms of micronutrients than macronutrients. Taking into account the RDA [37], the vast majority of the requirements for minerals and vitamins are achieved. On the other hand, it would be necessary to look for other recipes that included a little more protein and lipids to make it a nutritionally complete menu. Other recipes should be investigated, involving other types of nutrients that are more bioavailable or accessible to the human body.

### 3.3. Nutritional Comparison between Wild and Crop Plants

Table 4 shows some nutritional differences, for couples of species –wild taxa considered in this study and phylogenetically related cultivated ones, constituting ordinary agricultural crops—used in the same way as if they were the same plant. All data for the cultivated species have been recovered from CESNID’s (Center for Higher Education in Nutrition and Dietetics) Food Composition Tables [34].

Overall, for all nine couples of taxa, the wild species are richer in nutrients than the cultivated ones. For some nutrients, though, the difference between both species is striking, although it should be noted that some of the reported compounds may vary depending on several factors (seasonality, agronomic conditions, etc.). These comparisons clearly show that wild plants are by no means less interesting than their cultivated relatives in terms of nutritional values, and, ultimately, their consumption may be highly recommended.

### 3.4. WFPs as Folk Functional Foods

As we mentioned in the previous work, which is the basis of this one [8], many food plants (including WFPs) have been reported to also have medicinal uses, i.e., nutraceuticals [68,69] or folk functional foods [13]. It is well known that eating various green edible plants promotes health and longevity [70,71] and by eating mixtures of WFPs, humans increase their chance to obtain at least little of each health-promoting compound in plants [70]. Recently, Fantasma et al. [72] have reviewed the nutraceutical properties and chemical compounds of 10 plant taxa from the Apenines. A total of 6 out of these 10 plants are included in the present study at a specific level (exact coincidence), and one more at a generic level (different species of the same genus), reinforcing its role as food medicines.

### 3.5. Food Securtiy and Safety Precaution

It is commonly accepted by scientific knowledge that many plants—wild and cultivated—and other food constituents generally promote health at small natural concentrations, but are unhealthy at high concentrations [70]. In general, plants are not harmless and, in some cases, wild food plants can contain antinutritional and potentially toxic compounds such as oxalic acid, pyrrolizidine and pyrrolidine alkaloids, brydiofin, cucurbitacins, saponins, phenanthrenes and hydroxyanthracenes, monoterpenes, phenylpropanoids or prenylflavonoids [73]. In addition, in some cases, toxic or adverse effects are identified but responsible compounds are unknown. In order to evaluate the safety of the plants for human consumption reported in this research, the European Food Safety Authority (EFSA) [74] list has been consulted. In total, 33 from 93 taxa appear in the compendium of botanicals reported to contain naturally occurring substances of possible concern for human health when used in food and food supplements, always depending on the doses (Supplementary materials, Table S7). In any case, we must state that some of these plants are used daily in many diets, such as *Borago officinalis*, *Castanea sativa*, *Coriandrum sativum*, *Foeniculum vulgare*, *Juglans regia*, *Laurus nobilis*, *Origanum vulgare* and *Punica granatum*, among others, so that their regular use as edibles is supported by the practical evidence.

However, although precaution is necessary, as per the above-commented data, the evidence is that nutritional facts indicate that these plants are edible, thus confirming generations of ethnobotanical experience. A directive of the European Parliament admits, as a means for registering plant-based medicinal products, the evidence of their traditional use for the claimed effect for 30 years, 15 of them in the European Union [75]. Similarly, there is evidence of safe old-date-based food use of one plant, which is also confirmed here by nutritional data. Of course, as for all kinds of food (wild, cultivated, domesticated), safety is also linked to prudent consumption, which should be supposed in any case.

In addition, is necessary to know that, to collect wild plants, it is necessary to follow good practices in order to consume them cautiously. Only plants that are clearly identified and healthy must be gathered, trying to avoid areas contaminated and/or with pesticides or other polluting and potentially toxic compounds. In fact, it is necessary to exert the same precautions applied to cultivated plants (as per contamination) adding to precise identification, which is assured in market-sold crops.

## 4. Concluding Remarks

The data analyzed in this report demonstrate the wide existing variety of edible wild plants in the territory considered (this being extrapolatable elsewhere) and the nutritional richness that they bring into the ambit of dietetics. It has been shown that these plants are not only safe to eat, but also provide significant amounts of nutrients that are beneficial to the health of the human body, presenting as folk or traditional functional foods.

It is important to know the benefits of using these plants, which, in addition to having a more concentrated nutritional content, are easy to grow, since they grow in the wild. In addition, they contribute to the preservation of local knowledge on wild food taxa, including close relatives of landraces and other crops. Therefore, they are not only beneficial in terms of diet, but also in terms of traditional knowledge and associated genetic resources.

In fact, there is currently work in progress with, as targets, both research and further application also related to WFPs in the CLA. The Col·lectiu Eixarcolant’s program (www.eixarcolant.cat) aims at promoting a more sustainable, ethical and fair model of food production, distribution and consumption, and of socio-economic development, using as a tool the recovery of edible wild species and traditional agricultural landraces detected through ethnobotanical prospection. That is why they have taken the role of starting to market and distribute seeds of species such as *Chenopodium album* L., *Cynara cardunculus* L., *Portulaca oleracea* L., *Silene vulgaris* (Moench) Garke. and *Silybum marianum* L., all of them examined in this paper.

From the results of the present paper, it appears that an important number of traditionally well-known WFPs have relevant potential as food as per their nutritional composition.

However, extensive research is still needed on the nutritional content of certain plants that have been reported in several ethnobotanical works and lack or have very partial nutritional information. It is remarkable that, from the total of 153 taxa here prospected, only 80 have been decently and specifically examined from this viewpoint. Numerous taxa, 60, are still yet to be subject to a chemical or bromatological study. A further exploration, then, is necessary and encouraged in order to promote the distribution and consumption of these species, which have significant potential in terms of nutrition, sustainability and biocultural heritage conservation.

## Figures and Tables

**Table 1:** Main present micronutrients, their respective dominant taxa with the maximum value, the nutritional value per 100 g in fresh weight and the percentage of dietary reference intake. The information has been recovered from [26,29,34,38,39,40].

Nutrient	Taxa	Nutritional Value per 100 g (% of Dietary Reference Intake) [41]
Dietary fiber	*Satureja hortensis* L.	45.7 g (182.8%)
K	*Allium roseum* L.	1530 mg (43.8%) *
Na	*Capparis spinosa* L.	2960 mg (197.3%)
Ca	*Urtica urens* L.	4421 mg (442.1%) *
Fe	*Salvia officinalis* L.	84 mg (600%)
Mg	*Satureja hortensis* L.	377 mg (100.5%)
Mn	*Urtica urens* L.	10,400 μg (63,466.6%) *
Cu	*Chondrilla juncea* L.	430 μg (430%)
P	*Pinus pinea* L.	650 mg (92.8%)
Zn	*Corylus avellana* L.	2500 μg (16,666.6%)
Vitamin A	*Anethum graveolens* L.	7720 IU (28.5%)
Vitamin B1	*Coriandrum sativum* L.	239 μg (21.7%)
Vitamin B2	*Plantago major* L.	300 μg (21.4%)
Vitamin B3	*Coriandrum sativum* L.	2130 μg (13.3%)
Vitamin B6	*Carum carvi* L.	360 μg (25.7%)
Vitamin B9	*Allium ampeloprasum* L.	327 μg (81.8%)
Vitamin C	*Allium roseum* L.	1523 mg (1904%) *
Vitamin E (tocopherol equivalents)	*Artemisa absinthium* L.	2740 mg (18,267%) *
Vitamin K	*Rorippa nasturtium-aquaticum* L.	250 μg (333.3%)

* Expressed over 100 g of dry weight basis.

**Table 2:** Traditional preparation forms involving plants considered in the present paper.

Traditional Preparation	Synthetic Recipe Explanation	Researched Plants Involved
Cake	Basic preparation of the cake with flour, butter or olive oil and water (and sugar or salt depending on the kind of cake), and addition of the plant inside or on the surface	*Fragaria vesca* L., *Papaver rhoeas* L., *Silene vulgaris* L.
Condiment	Used to season a salad, a fritter, a pizza or a meat or fish roast or stew	*Laurus nobilis* L., *Origanum vulgare* L.
Fritter	Put in batter and fried in olive oil, with salt or sugar depending on the use as meat dishes’ complement or as a dessert	*Sambucus nigra* L.
Omelette	Slightly fried in olive oil or sauté, mixed with eggs, and fried in olive oil	*Borago officinalis* L.
Preserve	Cooked in sugar, preserved in brine or preserved in vinegar	*Arbutus unedo* L., *Foeniculum vulgare* Mill., *Rubus ulmifolius* Schott.
Salad	Cleaned and seasoned with olive oil and sometimes salt and/or condiments	*Molospermum peloponnesiacum* L., *Portulaca oleracea* L.
Soup	Boiled with water, in some cases with bread and an egg added at the end of the preparation	*Mentha spicata* L.
Stew	Stewed with meat	*Capparis spinosa* L., *Borago officinalis* L.
Sweet delicacy	Cleaned and directly consumed, often in the field just after collection	*Arbutus unedo* L., *Celtis australis* L., *Fragaria vesca* L., *Rubus idaeus* L., *Rubus ulmifolius* Schott.
Vegetable	Boiled in water	*Silene vulgaris* L.

**Table 3:** Nutritional value of the designed menu. Data are given in fresh weight.

Energy (kcal)		2036.1
Available carbohydrates (g)		339.6
Proteins (g)		31.1
Lipids (g)		25.5
Dietary fiber (g)		117.9
Main minerals		
	K (mg)	5285.1
	Na (mg)	352.0
	Ca (mg)	1165.8
	Fe (mg)	14.4
	Mg (mg)	434.9
Main vitamins		
	Vitamin B9 (μg)	3700
	Vitamin C (mg)	1585.2
	Vitamin E (tocopherol equivalents) (mg)	28.9
	Vitamin K (μg)	221.3

**Table 4:** Differences within wild and crop plants used as the same plant [26,28,34,67]. All data are given per 100 g in fresh weight. * The value rounded to one decimal is close to 0.

Taxon	Energy (kcal)	Available Carbohydrates (g)	Proteins (g)	Lipids (g)	Dietary Fiber (g)	K (mg)	Na (mg)	Ca (mg)	Fe (mg)	Mg (mg)	Vitamin A (IU)	Vit. B9 (μ g)	Vit. C (mg)	Vit. E (Tocopherol Equivalents) (mg)
*Allium ampeloprasum* L. (wild)	85	16.6	1.7	0.3	4.2	455	32.7	75.6	0.5	17.1	-	145	6.7	0.0 *
*Allium sativum* L. (crop)	119	23.4	5.7	0.4	2.1	446	53	25	1.3	23	-	3	22	0.1
*Apium nodiflorum* Koch (wild)	21	1.2	1.6	0.4	2.7	165	244	152	1.8	28	-	125	-	2.6
*Apium graveolens* L. (crop)	10	1.5	0.9	0.1	2	305	110	52	0.5	14	0.0 *	36	8	0.2
*Asparagus acutifolius* L. (wild)	40	3.5	2.4	0.6	4.8	585	18.5	54.1	0.7	36.6	-	217	37.8	83
*Asparagus officinalis* L. (crop)	21	2.7	2.9	0.2	0.6	269	3	28	1.3	11	-	52	1.4	1.7
*Beta vulgaris* L. subsp. *maritima* (L.) Arcang. (wild)	31	1.7	3.1	0.3	4.4	988	201	67.1	2.9	66.9	-	300	36.4	0.5
*Beta vulgaris* L. subsp. *vulgaris* (crop)	21	2.7	2.1	0.2	1	378	170	80	2.3	81	0.2	14	35	0.0 *
*Cichorium intybus* L. (wild)	33	3.5	1.8	0.5	3.6	299	70.8	153	1.3	19.8	-	3253	19.7	-
*Cihorium endivia* L. (crop)	12	1	1.6	0.2	2.6	327	14	55	1	12	-	0.2	10	-
*Eruca vesicaria* L. (wild)	28	2.1	2.6	0.7	1.6	413	14.1	250	1.8	33.7	-	-	125	-
*Eruca sativa* Mill. (crop)	25	3.6	2.6	0.6	1.6	369	27	160	1.5	47	-	-	15	-
*Fragaria vesca* L. (wild)	32	7.7	0.6	0.3	2	153	-	16	0.4	-	3.3	240	58.8	-
*Fragaria chiloensis* L. (crop)	27	5.5	0.6	0.3	1.6	152	-	21	0.4	-	2	0.1	57	-
*Lactuca perennis* L. (wild)	32.4	4.4	2.7	0.4	6.5	440.8	47.4	331.1	3.3	31.6	-	-	-	-
*Lactuca sativa* L. (crop)	16	1.7	1.4	0.4	1.5	234	22	40	0.6	10	-	-	-	-
*Vaccinium myrtillus* L. (wild)	33	6.1	0.6	0.6	4.9	78	1	10	0.7	2.4	19	-	22	1.9
*Vaccinium macrocarpon* Aiton (crop)	48	11.3	0.6	-	3	68	2	9	0.5	4	0.0 *	1	20	0.3

## Data Availability

The original contributions presented in the study are included in the article or supplementary material, further inquiries can be directed to the corresponding author.

## Supplementary Materials

The following supporting information can be downloaded at: https://www.mdpi.com/article/10.3390/foods13172785/s1, Table S1: General nutritional data for each taxon; Table S2: Minerals and nitrates data for each taxon; Table S3: Carotenoids, vitamins and phenols data for each taxon; Table S4: General nutritional data from species of the same genus as some plants quoted in this paper lacking such information, and for the genus in case of *Rosa* sp.; Table S5: Minerals and nitrates data from species of the same genus as some plants quoted in this paper lacking such information, and for the genus in case of *Rosa* sp.; Table S6: Carotenoids, vitamins and phenols data from species of the same genus as some plants quoted in this paper lacking such information, and for the genus in case of *Rosa* sp.; Table S7: Taxa with nutritional data reported in this research and considered for the European Food Safety Authority for their possible chemical concern or toxic/adverse effects, depending on the doses [76,77,78,79,80,81,82,83,84,85,86,87,88,89,90,91,92,93,94,95,96].
