# Supplementary material for: Nutritional Properties of Wild Edible Plants with Traditional Use in the Catalan Linguistic Area: A First Step for Their Relevance in Food Security
Source: Foods. 2024 Sep 1;13(17):2785. doi: 10.3390/foods13172785 (PMC11394892; doi:10.3390/foods13172785)
Supplement: Supplementary file 1 [file foods-13-02785-s001.zip › foods-3173831-supplementary.pdf]

Table S1. General nutritional data for each taxon. All data are given per 100 g in fresh weight.

Taxon	Family	Part of the plant	Energy (kcal)	Available carbohydrates (g)	Proteins (g)	Lipids (g)	Dietary fiber (g)	Ref.
Allium ampeloprasum L.	Amaryllidaceae	fresh bulb	85	16.6	1.7	0.3	4.2	1
Allium roseum L.	Amaryllidaceae	dry bulb	250.4	318	22.7	3.6	12.3	2
Amelanchier ovalis Medic.	Rosaceae	fresh fruit	84.9	18.5	3.2	1.9	6	3
Anethum graveolens L.	Apiaceae	fresh leaf	43	7	3.5	1.1	2.1	4
Aphyllanthes monspeliensis L.	Asparagaceae	dry flower	-	-	5.7	1.2	-	5
Apium nodiflorum Koch	Apiaceae	fresh leaf	21	1.2	1.6	0.4	2.7	1
Arbutus unedo L.	Ericaceae	fresh fruit	167	30.4	1.1	0.6	17.3	1
Artemisia absinthium L.	Asteraceae	dry leaf	87.9	8.3	-	6.1	14.2	6
Artemisia alba Turra	Asteraceae	dry leaf	-	0.1	0.1	-	0.4	7
Arundo donax L.	Poaceae	fresh shoot	369.7	82.6	6.9	1.3	37.5	8
Asparagus acutifolius L.	Asparagaceae	fresh shoot	40	3.5	2.4	0.6	4.8	1
Asparagus officinalis L.	Asparagaceae	fresh shoot	25	5	3	0.4	-	9
Beta vulgaris L. subsp. *maritima* (L.) Arcang.	Amaranthaceae	fresh leaf	31	1.7	3.1	0.3	4.4	1
Borago officinalis L.	Boraginaceae	fresh leaf	44	9.5	1.2	0.2	-	1
Bryonia cretica L. subsp. *dioica* (Jacq.) Tutin	Cucurbitaceae	fresh fruit	55	4.2	4	1.1	4.6	1
Capparis spinosa L.	Capparaceae	fresh flower	20	5	2	0.9	3	7
Carum carvi L.	Apiaceae	fresh seed	333	-	19.8	14.6	38	4
Castanea sativa Mill.	Fagaceae	fresh fruit	175	49.9	3.2	1.9	6	10
Celtis australis L.	Cannabaceae	dry fruit	-	-	4.4	-	13.3	11
Chamaerops humilis L.	Arecaceae	fresh fruit	-	-	30.3	1.1	18	12
Chenopodium album L.	Amaranthaceae	fresh leaf	53	5.9	2.7	0.6	6.4	1
Chenopodium ambrosioides L.	Amaranthaceae	fresh leaf	32	7.4	0.3	0.5	3.8	4
Chondrilla juncea L.	Asteraceae	fresh leaf	44	3.6	2.5	0.8	7.7	1
Cichorium intybus L.	Asteraceae	fresh leaf	33	3.5	1.8	0.5	3.6	1
Coriandrum sativum L.	Apiaceae	fresh fruit	298	55	12.2	17.8	41.9	13
Corylus avellana L.	Betulaceae	fresh seed	639	7.2	13	62	7.5	14
Crataegus monogyna Jacq.	Rosaceae	fresh fruit	139	25.9	1.4	0.6	11.8	1
Crepis vesicaria L.	Asteraceae	fresh leaf	41.8	3.8	1	0.7	4.2	15
Crithmum maritimum L.	Apiaceae	fresh leaf	30	2.1	2.2	0.4	4.7	1
Cynara cardunculus L.	Asteraceae	fresh flower	22	5.3	0.8	0.1	1.7	4
Cytinus hypocistis L.	Cytinaceae	dry fruit	382.4	86.6	4.9	0.7	4.8	16
Eruca vesicaria L.	Brassicaceae	fresh leaf	28	2.1	2.6	0.7	1.6	1
Ficus carica L.	Moraceae	fresh fruit	350.3	20	1.2	0.4	-	9, 17
Foeniculum vulgare Mill.	Apiaceae	fresh leaf	63	9.7	2.8	0.4	3.9	1
Fragaria vesca L.	Rosaceae	fresh fruit	32	7.7	0.7	0.3	2	4
Globularia alypum L.	Plantaginaceae	dry leaf	240.5	47.1	6.2	3.1	-	18
Helianthus tuberosus L.	Asteraceae	fresh tuber	73	17.4	2	-	1.6	4
Juglans regia L.	Juglandaceae	fresh fruit	650	16	20	60	6	1, 19
Lactuca perennis L.	Asteraceae	fresh leaf	32.5	4.4	2.7	0.5	6.4	20
Laurus nobilis L.	Lauraceae	fresh leaf	313	75	7.6	8.4	26.3	4
Malva sylvestris L.	Malvaceae	fresh leaf	35	2.2	3	0.6	4.8	1
Medicago sativa L.	Fabaceae	fresh leaf	52	14	6	1.2	-	9
Mentha pulegium L.	Lamiaceae	fresh leaf	-	-	-	-	-	21
Mentha spicata L.	Lamiaceae	fresh leaf	44	8.4	3.3	0.7	6.8	4
Molopospermum peloponnesiacum L.	Apiaceae	fresh leaf	45	5.6	3.3	-	4.7	22

Myrtus communis L.	Myrtaceae	fresh leaf	93	24	0.8	0.7	-	1
Opuntia maxima Mill.	Cactaceae	fresh fruit	16	3.3	1.3	0.1	2.2	23
Origanum vulgare L.	Lamiaceae	fresh leaf	265	69	9	4.3	42.5	4
Papaver rhoeas L.	Papaveraceae	fresh leaf	42	3.4	3.5	0.6	4.4	1
Pinus pinea L.	Pinaceae	fresh fruit	678	2.9	14	67.8	8.5	14
Plantago lanceolata L.	Plantaginaceae	fresh leaf	28	2.8	1.7	0.3	3.7	1
Plantago major L.	Plantaginaceae	fresh leaf	61	15	2.5	0.3	-	1
Portulaca oleracea L.	Portulacaceae	fresh leaf	16	3.4	1.3	0.1	-	24
Prunus spinosa L.	Rosaceae	fresh fruit	114	19.7	1.2	0.5	12.3	1
Punica granatum L.	Lythraceae	fresh fruit	61	13.7	1	0.3	3.5	1
Ribes alpinum L.	Grossulariaceae	fresh fruit	50	12	1.4	0.5	-	9
Ribes nigrum L.	Grossulariaceae	fresh fruit	65	16	1.3	0.4	-	9
Rorippa nasturtium-aquaticum L.	Apiaceae	fresh leaf	25	4.5	0.9	0.2	1.8	1
Rosmarinus officinalis L.	Lamiaceae	fresh leaf	131	20.7	3.3	5.9	14.1	4
Rubus idaeus L.	Rosaceae	fresh fruit	38	7	1.2	0.5	6.7	4
Rubus ulmifolius Schott.	Rosaceae	fresh fruit	99	16.1	1.7	0.5	11.5	1
Rumex acetosa L.	Polygonaceae	fresh leaf	33	2	2.4	0.7	4.4	1
Rumex acetosella L.	Polygonaceae	fresh leaf	30	4	2	0.1	1.4	25
Salvia officinalis L.	Lamiaceae	fresh leaf	315	57.9	17	3.5	36	26
Sambucus nigra L.	Caprifoliaceae	fresh fruit	73	18.4	0.7	0.5	7	1
Sanguisorba minor Scop.	Rosaceae	dry leaf	46	2.9	5.2	1.5	-	27
Satureja hortensis L.	Lamiaceae	fresh leaf	272	68.7	6.7	5.9	45.7	4
Satureja montana L.	Lamiaceae	fresh leaf	-	-	-	-	-	4
Scorzonera hispanica L.	Asteraceae	fresh leaf	70	18	4	0.4	-	28
Silene vulgaris (L.) Moench	Caryophyllaceae	fresh leaf	34	2.3	2.5	0.7	4.4	1
Silybum marianum L.	Asteraceae	fresh leaf	13	1.1	0.6	0.1	2.5	1
Sonchus oleraceus L.	Asteraceae	fresh leaf	33	2.3	2.2	0.6	3.4	1
Taraxacum officinale L.	Asteraceae	fresh leaf	44	3.6	2.5	0.8	7.7	9
Teucrium polium L.	Lamiaceae	fresh leaf	35	6.9	1.6	0.1	-	29
Urospermum dalechampii L.	Asteraceae	fresh leaf	33.1	5.4	2.2	0.3	-	30
Urtica membranacea L.	Urticaceae	fresh leaf	51	5	4.3	0.9	3	1
Urtica urens L.	Urticaceae	dry leaf	-	-	-	-	-	31
Vaccinium myrtillus L.	Ericaceae	fresh fruit	33	6.1	0.6	0.6	4.9	14
Valerianella locusta L.	Caprifoliaceae	fresh leaf	16	0.5	2	0.5	2.3	4
Vicia sativa L.	Fabaceae	dry seed	4410	1.8	28.3	1.3	4.8	1

Table S2. Minerals and nitrates data for each taxon. All data are given per 100 g in fresh weight.

Taxon	Family	Part of the plant	K (mg)	Na (mg)	Ca (mg)	Fe (mg)	Mg (mg)	Mn (μg)	Cu (μg)	P (mg)	Zn (μg)	Nitrates (mg)	Ref.
Allium ampeloprasum L.	Amaryllidaceae	fresh bulb	455	32.7	75.6	0.5	17.1	110	110	_	752	_	1
Allium roseum L.	Amaryllidaceae	dry bulb	1530	45.9	712.5	10.1	101.9	542	_	_	_	_	2
Amelanchier ovalis Medic.	Rosaceae	fresh fruit	500	9	40	0.9	33	_	_	74	2000	_	3
Anethum graveolens L.	Apiaceae	fresh leaf	738	61	208	6.6	55	1.3	_	_	910	_	4
Aphyllanthes monspeliensis L.	Asparagaceae	dry flower	_	_	_	_	_	_	_	_	_	_	5
Apium nodiflorum Koch	Apiaceae	fresh leaf	165	244	152	1.8	28	290	_	_	_	_	1
Arbutus unedo L.	Ericaceae	fresh fruit	334	20	111	0.9	9.7	146	99	32.5	_	_	1
Artemisia absinthium L.	Asteraceae	dry leaf	_	_	_	_	_	_	_	_	_	_	6
Artemisia alba Turra	Asteraceae	dry leaf	_	_	_	_	_	_	_	_	_	_	7
Arundo donax L.	Poaceae	fresh shoot	_	_	_	_	_	_	_	_	_	_	8
Asparagus acutifolius L.	Asparagaceae	fresh shoot	585	18.5	54.1	0.7	36.6	410	105	_	1059	_	1
Asparagus officinalis L.	Asparagaceae	fresh shoot	305	2	22	1	18	_	_	_	_	_	9
Beta vulgaris L. subsp. *maritima* (L.) Arcang.	Amaranthaceae	fresh leaf	988	201	67.1	2.9	66.9	730	_	_	_	200	1
Borago officinalis L.	Boraginaceae	fresh leaf	567	43.5	344	_	_	_	_	_	_	69.2	1
Bryonia cretica L. subsp. *dioica* (Jacq.) Tutin	Cucurbitaceae	fresh fruit	487	27.6	53.3	0.7	28.8	250	220	_	890	_	1
Capparis spinosa L.	Capparaceae	fresh flower	40	2960	40	1.7	33	78	_	_	_	_	7
Carum carvi L.	Apiaceae	fresh seed	_	_	689	_	_	_	_	_	_	_	4
Castanea sativa Mill.	Fagaceae	fresh fruit	500	9	40	0.9	33	_	_	74	500	_	10
Celtis australis L.	Cannabaceae	dry fruit	1060	_	269	5.1	_	_	_	143	710	_	11
Chamaerops humilis L.	Arecaceae	fresh fruit	1092.5	1.7	62.3	3.7	111.3	_	_	_	_	_	12
Chenopodium album L.	Amaranthaceae	fresh leaf	1155	9	307	5.3	207	1070	_	_	1300	_	1
Chenopodium ambrosioides L.	Amaranthaceae	fresh leaf	470	80	275	1.9	121	3098	_	_	_	_	4
Chondrilla juncea L.	Asteraceae	fresh leaf	1015	16.5	230	4.4	70.4	970	430	12.7	_	_	1
Cichorium intybus L.	Asteraceae	fresh leaf	299	70.8	153	1.3	19.8	260	_	_	370	12.9	1
Coriandrum sativum L.	Apiaceae	fresh fruit	1267	35	709	16.3	330	_	_	409	470	_	13
Corylus avellana L.	Betulaceae	fresh seed	502	18	194	3.4	236	_	_	_	2500	_	14
Crataegus monogyna Jacq.	Rosaceae	fresh fruit	459	_	235	1.8	83.3	742	179	_	311	_	1
Crepis vesicaria L.	Asteraceae	fresh leaf	591.3	76.8	309.9	0.6	45.5	830	_	_	_	_	15
Crithmum maritimum L.	Apiaceae	fresh leaf	252	464	224	2.3	76.6	990	_	_	_	63	1
Cynara cardunculus L.	Asteraceae	fresh flower	393	176	72	0.7	_	_	_	_	_	_	4
Cytinus hypocistis L.	Cytinaceae	dry fruit	_	_	_	_	_	_	_	_	_	_	16
Eruca vesicaria L.	Brassicaceae	fresh leaf	413	14.1	250	1.8	33.7	389	63.7	_	429	257	1
Ficus carica L.	Moraceae	fresh fruit	534.2	14.1	94.4	7.8	17	_	_	29	_	_	9, 17
Foeniculum vulgare Mill.	Apiaceae	fresh leaf	753	89.6	365	2.2	85	881	225	_	799	24	1
Fragaria vesca L.	Rosaceae	fresh fruit	153	_	16	0.4	13	380	_	_	_	_	4
Globularia alypum L.	Plantaginceae	dry leaf	481	30.2	2400	_	_	_	_	44.5	_	_	18
Helianthus tuberosus L.	Asteraceae	fresh tuber	429	_	14	3.4	17	60	_	_	_	_	4
Juglans regia L.	Juglandaceae	fresh fruit	500	10	100	3	169	_	_	380	_	_	1, 19
Lactuca perennis L.	Asteraceae	fresh leaf	440.8	47.4	331.1	3.3	31.6	_	_	47.4	_	_	20
Laurus nobilis L.	Lauraceae	fresh leaf	529	23	834	43	120	817	_	_	_	_	4
Malva sylvestris L.	Malvaceae	fresh leaf	692	-	226	3.6	-	-	_	_	_	_	1
Medicago sativa L.	Fabaceae	fresh leaf	-	-	120	5.4	-	-	_	_	_	_	9

Mentha pulegium L.	Lamiaceae	fresh leaf	-	-	-	-	-	-	-	-	-	-	21
Mentha spicata L.	Lamiaceae	fresh leaf	458	30	199	11.9	63	1120	-	-	-	-	4
Molopospermum peloponnesiacum L.	Apiaceae	fresh leaf	-	-	-	-	-	-	-	-	-	-	22
Myrtus communis L.	Myrtaceae	fresh leaf	-	-	-	-	-	-	-	-	-	-	1
Opuntia maxima Mill.	Cactaceae	fresh fruit	257	21	164	0.6	52	457	-	-	-	-	23
Origanum vulgare L.	Lamiaceae	fresh leaf	1260	25	1597	36.8	270	4990	-	-	-	-	4
Papaver rhoeas L.	Papaveraceae	fresh leaf	562	61	197	3.5	35.9	727	-	-	978	-	1
Pinus pinea L.	Pinaceae	fresh fruit	780	1	11	5.6	270	-	-	650	6.5	-	14
Plantago lanceolata L.	Plantaginaceae	fresh leaf	361	21.3	304	3.9	52.6	661	-	-	-	-	1
Plantago major L.	Plantaginaceae	fresh leaf	275	16	184	-	-	-	-	1.2	-	-	1
Portulaca oleracea L.	Portulacaceae	fresh leaf	494	45	65	2	68	303	-	-	-	-	24
Prunus spinosa L.	Rosaceae	fresh fruit	453	22.8	45.3	0.8	22.6	117	166	-	163	-	1
Punica granatum L.	Lythraceae	fresh fruit	247	4	13	1	6	-	-	25	-	-	1
Ribes alpinum L.	Grossulariaceae	fresh fruit	260	2	32	1	13	-	-	-	-	-	9
Ribes nigrum L.	Grossulariaceae	fresh fruit	325	2	55	1.6	24	-	-	-	-	-	9
Rorippa nasturtium-aquaticum L.	Apiaceae	fresh leaf	276	12	175	1.7	24.5	-	-	-	93.2	-	1
Rosmarinus officinalis L.	Lamiaceae	fresh leaf	668	26	317	6.7	91	960	-	-	-	-	4
Rubus idaeus L.	Rosaceae	fresh fruit	220	3	22	0.7	20	-	-	-	300	-	4
Rubus ulmifolius Schott.	Rosaceae	fresh fruit	196	32	78.4	1.2	43	1560	268	-	409	-	1
Rumex acetosa L.	Polygonaceae	fresh leaf	351	25.6	60.3	1	45	750	80	-	360	-	1
Rumex acetosella L.	Polygonaceae	fresh leaf	377	3	47	5	33	-	-	82	-	-	25
Salvia officinalis L.	Lamiaceae	fresh leaf	34.6	19.9	894	84	19.6	3133	757	-	4700	-	26
Sambucus nigra L.	Caprifoliaceae	fresh fruit	280	6	38	1.6	5	-	-	-	-	-	1
Sanguisorba minor Scop.	Rosaceae	dry leaf	932	2	985	-	-	-	-	-	-	-	27
Satureja hortensis L.	Lamiaceae	fresh leaf	1051	24	2132	37.9	377	6100	-	-	-	-	4
Satureja montana L.	Lamiaceae	fresh leaf	-	-	-	-	-	-	-	-	-	-	4
Scorzonera hispanica L.	Asteraceae	fresh leaf	320	5	53	3.3	-	-	-	-	-	-	28
Silene vulgaris (L.) Moench	Caryophyllaceae	fresh leaf	601	22.4	160	1.9	50.4	709	144	44.2	408	178	1
Silybum marianum L.	Asteraceae	fresh leaf	718	80.9	132	0.5	17.3	100	80.5	-	260	113	1
Sonchus oleraceus L.	Asteraceae	fresh leaf	481	-	131	2.9	43.3	881	249	-	597	100	1
Taraxacum officinale L.	Asteraceae	fresh leaf	1015	16.5	230	4.4	70.4	970	-	-	1630	-	9
Teucrium polium L.	Lamiaceae	fresh leaf	-	-	-	-	-	-	-	-	-	-	29
Urospermum dalechampii L.	Asteraceae	fresh leaf	344.5	42	153.7	2.5	14.3	-	-	24.2	-	-	30
Urtica membranacea L.	Urticaceae	fresh leaf	391	14.9	625	5.4	171	1698	350	111	839	-	1
Urtica urens L.	Urticaceae	dry leaf	3084	61	4421	83.9	-	10400	-	-	-	-	31
Vaccinium myrtillus L.	Ericaceae	fresh fruit	78	1	10	0.7	2.4	-	-	-	-	-	14
Valerianella locusta L.	Caprifoliaceae	fresh leaf	330	5	41	0.4	18	300	-	-	-	-	4
Vicia sativa L.	Fabaceae	dry seed	11	-	5.5	1.4	1.6	200	200	-	400	-	1

Table S3. Carotenoids, vitamins and phenols data for each taxon. All data are given per 100 g in fresh weight.

Taxon	Family	Part of the plant	β-carotene (μg)	Total carotenoids (mg)	Vit. A (IU)	Vit. B1 (μg)	Vit. B2 (μg)	Vit. B3 (μg)	Vit. B6 (μg)	Vit. B9 (μg)	Vit. C (mg)	Vit. E (tocopherol equivalents) (mg)	Vit. K (mg)	Flavonoids (mg)[1]	Phenols (mg)[2]	Ref.
Allium ampeloprasum L.	Amaryllidaceae	fresh bulb	-	-	-	-	-	-	-	145	6.7	0.03	-	-	-	1
Allium roseum L.	Amaryllidaceae	dry bulb	-	242.3	-	-	-	-	-	-	1523	-	-	3.4	736.7	2
Amelanchier ovalis Medic.	Rosaceae	fresh fruit	-	-	28	-	-	-	-	141	50	1.2	-	-	-	3
Anethum graveolens L.	Apiaceae	fresh leaf	-	-	7720	-	-	1570	-	1502	-	-	-	-	-	4
Aphyllanthes monspeliensis L.	Asparagaceae	dry flower	-	-	-	-	-	-	-	-	-	-	-	-	-	5
Apium nodiflorum Koch	Apiaceae	fresh leaf	-	-	-	-	-	-	-	125	-	2.6	-	45.5	-	1
Arbutus unedo L.	Ericaceae	fresh fruit	320	-	-	-	-	-	-	-	185	3.1	-	1.1	-	1
Artemisia absinthium L.	Asteraceae	dry leaf	-	-	-	-	-	-	-	-	-	2740	-	-	-	6
Artemisia alba Turra	Asteraceae	dry leaf	-	-	-	-	-	-	-	-	-	-	-	-	-	7
Arundo donax L.	Poaceae	fresh shoot	-	-	-	-	-	-	-	-	-	-	-	-	-	8
Asparagus acutifolius L.	Asparagaceae	fresh shoot	769	-	-	-	-	-	-	217	37.8	8.3	-	-	-	1
Asparagus officinalis L.	Asparagaceae	fresh shoot	-	-	900	-	-	-		-	33	-	-	-	-	9
Beta vulgaris L. subsp. *maritima* (L.) Arcang.	Amaranthaceae	fresh leaf	-	14.3	-	-	-	-	-	109	36.4	0.5	-	-	-	1
Borago officinalis L.	Boraginaceae	fresh leaf	2.9	-	-	-	-	-	-	-	1.8	1.2	-	18.9	-	1
Bryonia cretica L. subsp. *dioica* (Jacq.) Tutin	Cucurbitaceae	fresh fruit	-	-	-	-	-	-	-	-	-	-	-	-	-	1
Capparis spinosa L.	Capparaceae	fresh flower	83	-	138	-	-	-	-	-	4	-	-	-	-	7
Carum carvi L.	Apiaceae	fresh seed	-	-	363	-	-	-	360	-	21	-	-	-	-	4
Castanea sativa Mill.	Fagaceae	fresh fruit	-	-	28	-	-	-	-	10	50	1.2	-	-	-	10
Celtis australis L.	Cannabaceae	dry fruit	558	-	-	-	-	-	-	-	-	15.0	-	-	-	11
Chamaerops humilis L.	Arecaceae	fresh fruit	-	-	-	-	-	-	-	-	-	-	-	-	-	12
Chenopodium album L.	Amaranthaceae	fresh leaf	22.8	-	-	-	20	-	-	9	155	-	-	80	-	1
Chenopodium ambrosioides L.	Amaranthaceae	fresh leaf	38	-	57	-	-	-	-	210	3.6	-	-	-	-	4
Chondrilla juncea L.	Asteraceae	fresh leaf	-	2.1	-	-	-	-	-	-	16.5	0.6	-	7.4	-	1
Cichorium intybus L.	Asteraceae	fresh leaf	8.7	-	-	-	-	-	-	3253	19.7	1.0	173	-	-	1
Coriandrum sativum L.	Apiaceae	fresh fruit	-	-	-	239	290	2130	-	-	21	-	-	-	-	13
Corylus avellana L.	Betulaceae	fresh seed	-	0.3	-	-	-	-	-	113	1	25	-	-	-	14
Crataegus monogyna Jacq.	Rosaceae	fresh fruit	-	-	-	5	-	-	-	-	27.8	1.9	-	110	-	1
Crepis vesicaria L.	Asteraceae	fresh leaf	-	-	-	-	-	-	-	-	-	-	-	-	-	15
Crithmum maritimum L.	Apiaceae	fresh leaf	-	4.5	-	-	-	-	-	-	62	-	-	-	1260	1
Cynara cardunculus L.	Asteraceae	fresh flower	-	-	-	-	-	-	-	-	-	-	-	-	-	4
Cytinus hypocistis L.	Cytinaceae	dry fruit	-	-	-	-	-	-	-	-	-	-	-	-	-	16
Eruca vesicaria L.	Brassicaceae	fresh leaf	1280	-	-	40	90	300	70	-	125	-	31	-	211	1
Ficus carica L.	Moraceae	fresh fruit	-	-	120	-	-	-	-	-	5	-	-	-	-	9, 17
Foeniculum vulgare Mill.	Apiaceae	fresh leaf	1190	-	-	-	-	-	-	3273	66.7	1.7	239	-	195	1
Fragaria vesca L.	Rosaceae	fresh fruit	-	-	3.3	-	-	-	-	240	58.8	-	-	-	-	4

Globularia alypum L.	Plantaginceae	dry leaf	-	-	-	-	-	-	-	-	-	-	-	-	-	18
Helianthus tuberosus L.	Asteraceae	fresh tuber	12	-	20	-	-	-	-	13	4	0.2	-	-	-	4
Juglans regia L.	Juglandaceae	fresh fruit	-	-	120	-	-	-	-	-	3	-	-	-	-	1, 19
Lactuca perennis L.	Asteraceae	fresh leaf	-	-	-	-	-	-	-	-	-	0.9	-	-	-	20
Laurus nobilis L.	Lauraceae	fresh leaf	-	-	6185	-	-	-	-	180	46.5	-	-	-	-	4
Malva sylvestris L.	Malvaceae	fresh leaf	-	-	-	-	-	-	-	-	-	-	-	-	-	1
Medicago sativa L.	Fabaceae	fresh leaf	-	-	-	-	-	-	-	-	-	-	-	-	-	9
Mentha pulegium L.	Lamiaceae	fresh leaf	-	-	-	-	-	-	-	-	-	-	-	-	-	21
Mentha spicata L.	Lamiaceae	fresh leaf	-	-	4054	-	-	-	-	14	13.3	-	-	-	-	4
Molopospermum peloponnesiacum L.	Apiaceae	fresh leaf	-	-	-	-	-	-	-	-	-	-	-	-	-	22
Myrtus communis L.	Myrtaceae	fresh leaf	-	-	-	-	-	-	-	-	36	-	-	-	-	1
Opuntia maxima Mill.	Cactaceae	fresh fruit	250	-	457	-	-	-	-	-	9.3	-	-	-	-	23
Origanum vulgare L.	Lamiaceae	fresh leaf	1007	-	1791	-	-	-	-	200	2.3	18.3	-	-	-	4
Papaver rhoeas L.	Papaveraceae	fresh leaf	750	-	-	-	-	-	-	152	24	1	145	31	-	1
Pinus pinea L.	Pinaceae	fresh fruit	-	0.0*	3.3	-	-	-	-	34	-	13.6	-	-	-	14
Plantago lanceolata L.	Plantaginaceae	fresh leaf	-	6.8	-	-	-	-	-	-	13.6	-	-	-	-	1
Plantago major L.	Plantaginaceae	fresh leaf	-	-	1510	100	300	-	-	-	-	-	-	-	-	1
Portulaca oleracea L.	Portulacaceae	fresh leaf	-	-	1320	-	-	-	-	12	21	-	-	-	-	24
Prunus spinosa L.	Rosaceae	fresh fruit	330	-	-	-	-	-	-	-	10.5	5.2	-	-	-	1
Punica granatum L.	Lythraceae	fresh fruit	-	0.0*	23	-	-	-	-	38	20	0.5	-	-	-	1
Ribes alpinum L.	Grossulariaceae	fresh fruit	-	-	120	-	-	-	-	-	40	-	-	-	-	9
Ribes nigrum L.	Grossulariaceae	fresh fruit	-	-	230	50	40	-	-	-	180	-	-	-	-	9
Rorippa nasturtium-aquaticum L.	Apiaceae	fresh leaf	3710	-	-	-	-	470	-	-	53	1.5	250	-	-	1
Rosmarinus officinalis L.	Lamiaceae	fresh leaf	-	-	487	-	-	-	-	109	21.8	-	-	-	-	4
Rubus idaeus L.	Rosaceae	fresh fruit	-	0.0*	-	-	-	-	60	-	25	0.2	-	-	-	4
Rubus ulmifolius Schott.	Rosaceae	fresh fruit	380	-	-	-	-	-	-	-	23	3.4	-	29.6	-	1
Rumex acetosa L.	Polygonaceae	fresh leaf	-	-	-	-	-	-	-	187	25.8	1.3	-	39.5	-	1
Rumex acetosella L.	Polygonaceae	fresh leaf	-	-	-	-	-	-	-	100	6.7	0.0*	-	42.2	-	25
Salvia officinalis L.	Lamiaceae	fresh leaf	3480	-	5900	-	-	-	-	274	32.4	7.5	1.7	-	-	26
Sambucus nigra L.	Caprifoliaceae	fresh fruit	-	-	600	-	-	-	-	6	36	-	-	-	-	1
Sanguisorba minor Scop.	Rosaceae	dry leaf	-	-	-	-	-	-	-	-	-	-	-	-	-	27
Satureja hortensis L.	Lamiaceae	fresh leaf	-	-	5310	-	-	-	-	-	50	-	-	-	-	4
Satureja montana L.	Lamiaceae	fresh leaf	-	-		-	-	-	-	-	-	-	-	-	-	4
Scorzonera hispanica L.	Asteraceae	fresh leaf	-	-	6	100	40	-	-	-	4	-	-	-	-	28
Silene vulgaris (L.) Moench	Caryophyllaceae	fresh leaf	1030	-	-	-	-	-	-	200	25.5	10.1	172	-	-	1
Silybum marianum L.	Asteraceae	fresh leaf	-	-	-	-	-	-	-	41.7	2.7	0.0*	-	1.1	-	1
Sonchus oleraceus L.	Asteraceae	fresh leaf	1050	-	-	-	-	-	-	85.81	38.7	0.7	175	14.8	-	1
Taraxacum officinale L.	Asteraceae	fresh leaf	-	2.1	-	-	-	-	-	-	16.5	0.6	-	7.4	-	9
Teucrium polium L.	Lamiaceae	fresh leaf	-	0.0*	-	-	-	-	-	-	25.5	0.3	-	-	-	29
Urospermum dalechampii L.	Asteraceae	fresh leaf	2614	-	1453	-	-	-	-	-	-	-	-	-	-	30
Urtica membranacea L.	Urticaceae	fresh leaf	5720	-	-	20	230	620	70	-	285	-	-	-	-	1
Urtica urens L.	Urticaceae	dry leaf	-	-	-	-	-	-	-	-	-	-	-	-	-	31
Vaccinium myrtillus L.	Ericaceae	fresh fruit	-	-	19	-	-	-	-	-	22	1.9	-	-	-	14
Valerianella locusta L.	Caprifoliaceae	fresh leaf	3280	-	-	-	-	-	-	-	2.4	0.2	48.9	-	-	4
Vicia sativa L.	Fabaceae	dry seed	-	-	-	-	-	-	-	-	-	-	-	-	-	1

[1] Expressed as catechin (mg CAE / 100 mg); [2] Expressed as gallic acid (mg GAE / 100 mg); *The value rounded to one decimal is close to 0.

Table S4. General nutritional data from species of the same genus as some plants quoted in this paper lacking such information, and for the genus in case of *Rosa* sp. All data are given per 100 g in fresh weight.

Taxon	Family	Part of the plant	Energy (kcal)	Available carbohydrates (g)	Proteins (g)	Lipids (g)	Dietary fibre (g)	Ref.
Asparagus horridus L.	Asparagaceae	fresh shoot	32.5	4.3	2.7	0.5	4.8	1
Chenopodium bonus-henricus L.	Amaranthaceae	fresh leaf	42.5	6.7	1.5	0.6	5.1	1, 4
Lactuca serriola L.	Asteraceae	fresh leaf	32.5	4.4	2.7	0.5	6.4	20
Mentha aquatica L.	Lamiaceae	fresh leaf	44	8.4	3.3	0.7	6.8	4, 21
Mentha suaveolens Ehrh.	Lamiaceae	fresh leaf	44	8.4	3.3	0.7	6.8	4, 21
Ribes petraeum Wulfen in Jacq.	Grossulariaceae	fresh fruit	57.5	14	1.4	0.5	-	9
Rumex scutatus L.	Polygonaceae	fresh leaf	31.5	3	2.2	0.4	2.9	1, 25
Sonchus tenerrimus L.	Asteraceae	fresh leaf	33	2.3	2.2	0.6	3.4	1
Taraxacum dissectum Ledeb.	Asteraceae	fresh leaf	44	3.6	2.5	0.8	7.7	9
Urtica dioica L.	Urticaceae	fresh leaf	51	5	4.3	0.9	3	1, 31
Rosa sp.	Rosaceae	dry flower	-	-	-	-	-	32
Satureja calamintha (L.) Scheele	Lamiaceae	fresh leaf	272	68.7	6.7	5.9	45.7	4
Satureja fruticosa (L.) Briq.	Lamiaceae	fresh leaf	272	68.7	6.7	5.9	45.7	4

Table S5. Minerals and nitrates data from species of the same genus as some plants quoted in this paper lacking such information, and for the genus in case of *Rosa* sp. All data are given per 100 g in fresh weight.

Taxon	Family	Part of the plant	K (mg)	Na (mg)	Ca (mg)	Fe (mg)	Mg (mg)	Mn (µg)	Cu (µg)	P (mg)	Zn (µg)	Nitrates (mg)	Ref.
Asparagus horridus L.	Asparagaceae	fresh shoot	445	10.3	38.1	0.8	27.3	410	105	-	1059	-	1
Chenopodium bonus-henricus L.	Amaranthaceae	fresh leaf	812.5	44.5	291	3.6	164	2084	-	-	1300	-	1, 4
Lactuca serriola L.	Asteraceae	fresh leaf	440.8	47.4	331.1	3.3	31.6	-	-	47.4	-	-	20
Mentha aquatica L.	Lamiaceae	fresh leaf	458	30	199	11.9	63	1 120	-	-	-	-	4, 21
Mentha suaveolens Ehrh.	Lamiaceae	fresh leaf	458	30	199	11.9	63	1 120	-	-	-	-	4, 21
Ribes petraeum Wulfen in Jacq.	Grossulariaceae	fresh fruit	292.5	2	43.5	1.3	18.5	-	-	-	-	-	9
Rumex scutatus L.	Polygonaceae	fresh leaf	364	14.3	53.7	3	39	750	80	82	360	-	1, 25
Sonchus tenerrimus L.	Asteraceae	fresh leaf	481	-	131	2.9	43.3	881	249	-	1100	100	1
Taraxacum dissectum Ledeb.	Asteraceae	fresh leaf	1015	16.5	230	4.4	70.4	970	-	-	1630	-	9
Urtica dioica L.	Urticaceae	fresh leaf	391	14.9	625	5.4	171	1 698	350	111	839	-	1, 31
Rosa sp.	Rosaceae	dry flower	639	-	196	-	114	-	-	513	-	-	32
Satureja calamintha (L.) Scheele	Lamiaceae	fresh leaf	1051	24	2 132	37.9	377	6100	-	-	-	-	4
Satureja fruticosa (L.) Briq.	Lamiaceae	fresh leaf	1051	24	2132	37.9	377	6100	-	-	-	-	4

Table S6. Carotenoids, vitamins and phenols data from species of the same genus as some plants quoted in this paper lacking such information, and for the genus in case of *Rosa* sp. All data are given per 100 g in fresh weight.

Taxon	Family	Part of the plant	β-carotene (μg)	Total carotenoids (mg)	Vit. A (IU)	Vit. B1 (μg)	Vit. B2 (μg)	Vit. B3 (μg)	Vit. B6 (μg)	Vit. B9 (μg)	Vit. C (mg)	Vit. E (tocopherol equivalents) (mg)	Vit. K (μg)	Flavonoids (mg)[1]	Phenols (mg)[2]	Ref.
Asparagus horridus L.	Asparagaceae	fresh shoot	700	-	900	-	-	-	-	200	35.4	8.3	-	-	-	1
Chenopodium bonus-henricus L.	Amaranthaceae	fresh leaf	30.4	-	57	-	20	-	-	200	79.3	-	-	80	-	1, 4
Lactuca serriola L.	Asteraceae	fresh leaf	-	-	-	-	-	-	-	-		0.9	-	-	-	20
Mentha aquatica L.	Lamiaceae	fresh leaf	-	-	4054	-	-	-	-	-	13.3	-	-	-	-	4, 21
Mentha suaveolens Ehrh.	Lamiaceae	fresh leaf	-	-	4054	-	-	-	-	-	13.3	-	-	-	-	4, 21
Ribes petraeum Jacq.	Grossulariaceae	fresh fruit	-	-	175	50	40	-	-	-	110	-	-	-	-	9
Rumex scutatus L.	Polygonaceae	fresh leaf	-	-	-	-	-	-	-	200	16.2	0.7	-	40.9	-	1, 25
Sonchus tenerrimus L.	Asteraceae	fresh leaf	1050	-	-	-	-	-	-	100	38.7	0.7	175	14.8	-	1
Taraxacum dissectum Ledeb.	Asteraceae	fresh leaf	-	2.1	-	-	-	-	-	-	16.5	0.6	-	7.4	-	9
Urtica dioica L.	Urticaceae	fresh leaf	5720	-	-	20	230	620	70	-	285	-	-	-	-	1, 31
Rosa sp.	Rosaceae	dry flower	-	-	-	-	-	-	-	-		-	-	-	-	32
Satureja calamintha (L.) Scheele	Lamiaceae	fresh leaf	-	-	5310	-	-	-	-	-	50	-	-	-	-	4
Satureja fruticosa (L.) Briq.	Lamiaceae	fresh leaf	-	-	5310	-	-	-	-	-	50	-	-	-	-	4

[1] Expressed as catechin (mg CAE / 100 mg); [2] Expressed as gallic acid (mg GAE / 100 mg)

Table S7. Taxa with nutritional data reported in this research and considered for the European Food Safety Authority [25] for their possible chemical concern or toxic/adverse effects, depending on the doses.

Taxon	Family	Part of plant reported in our study	Parts of plants of possible concern	Chemicals of concern	Remarks on toxic/adverse effect(s) not known to be related to the identified chemical(s) of concern
Anethum graveolens L.	Apiaceae	fresh leaf	whole plant	Essential oil: phenylpropanoids: e.g. methylchavicol.	-
Artemisia absinthium L.	Asteraceae	dry leaf	aerial part	Essential oil of (Z)-epoxy-ocimene chemotype: bicyclic monoterpenes: e.g. alpha-thujone (up to 0.30%), beta-thujone (up to 7.78%), camphor (0.19-9.30%). Essential oil of sabinyl acetate chemotype: alpha-thujone (0.12-0.2%), beta-thujone (0.58-0.71%), camphor (up to 0.31%). Essential oil of chrysanthenyl acetate chemotype: alpha-thujone (1.32%), beta-thujone (18.72%), camphor (0.18%). Essential oil of beta-thujone chemotype: alpha-thujone (0.53-2.76%), beta-thujone (17.5-59.9%), camphor (0.10-0.16%). Essential oil of beta-thujone/epoxy ocimene mixed chemotypes: alpha-thujone (0.7-1.68%), beta-thujone (20.9-40.6%). Essential oil of cis-chrysanthenol chemotype: alpha-thujone 2.55-21.6%, beta-thujone (3.75-25.9%).	-
Arundo donax L.	Poaceae	fresh shoot	rhizome	Indole alkaloids: e.g. donasine.	-
Borago officinalis L.	Boraginaceae	fresh leaf	aerial part	Genus in which species may contain unsaturated pyrrolizidine alkaloids: e.g. lycopsamine, 7-acetyl-lycopsamine, amabiline, supinine.	-
Bryonia cretica L. subsp. *dioica* (Jacq.) Tutin	Cucurbitaceae	fresh fruit	whole plant	Genus in which species may contain oxygenated tetracyclic triterpene derivatives: e.g. cucurbitacines.	-
Carum carvi L.	Apiaceae	fresh seed	fruit	Essential oil: monoterpene ketone: e.g. (S)-(+)-carvone (50-65%).	-
Castanea sativa Mill.	Fagaceae	fresh fruit	aerial part	-	Hydrolysable tannins, e.g. ellagitannins used at high doses and over a long period may have a negative impact on liver.
Cheopodium album L.	Amaranthaceae	fresh leaf	leaf	Essential oil : peroxygenated monoterpene: ascaridole (45%).	-
Chenopodium ambrosioides L.	Amaranthaceae	fresh leaf	aerial part	Essential oil: peroxygenated monoterpene: ascaridole.	-
Coriandrum sativum L.	Apiaceae	fresh fruit	aerial part	Essential oil from the fruit: bicyclic monoterpene: camphor (3-9%).	-
Crithmum maritimum L.	Apiaceae	fresh leaf	leaf	Essential oil: phenylpropanoids: e.g. methylchavicol (3,4%).	-
Ficus carica L.	Moraceae	fresh fruit	whole plant	Furanocoumarins from latex: e.g. psoralen and bergapten.	-
Foeniculum vulgare Mill	Apiaceae	fresh leaf	aerial part	Essential oil from the aerial part: phenylpropanoids, e.g. trans-anethole, methylchavicol (2.3-4.9%)Essential oil from the unripe seed: methylchavicol (11.9-56.1%)Essential oil from ripe seed: methylchavicol (61.8%).	-
Globularia alypum L.	Plantaginaceae	dry leaf	leaf and root	-	Increased embryo resorption observed in pregnant rats after intragastric administration of 800 mg/kg of an ethanolic extract of the dried leaf from day 1-6 of pregnancy.
Juglans regia L.	Juglandaceae	fresh fruit	fruit, husk and	Naphthoquinones in fruit, husk and leaf: e.g. juglone.	Juglone is found in 29.8%

			leaf		of the surface waxes of the fruit (pericarp) and 28.6% of the surface waxes of the leaf.
Laurus nobilis L.	Lauraceae	fresh leaf	fruit and leaf	Essential oil from leaf: phenylpropanoids: e.g. methyleugenol (1.7-11.8%) and monoterpene etheroxide: 1,8-cineole (34-53%).	-
Medicago sativa L.		fresh leaf	aerial part and seed	Pyrrolidine alkaloids in the seed: e.g. stachydrine (0.18%), homostachydrine; and aromatic nitro-derivatives: e.g. trigonelline (0.36%).	-
Mentha pulegium L.	Lamiaceae	fresh leaf	aerial part	Essential oil: monocyclic monoterpene ketones: e.g. pulegone (71.3-90%), bicyclic monoterpenes: menthofuran, thujones and monoterpene etheroxide: 1,8-cineole.	-
Mentha spicata L.	Lamiaceae	fresh leaf	aerial part	Essential oil: monocyclic monoterpene ketone: e.g. pulegone (1.7-1.9%) and monoterpene etheroxide: 1,8-cineole (6-6.8%) Essential oil chemotype carvone: 1,8-cineole (0.5%) Essential oil chemotype dihydrocarvyl acetate: 1,8-cineole (2.2%).	-
Myrtus communis L.	Myrtaceae	fresh leaf	aerial part	Essential oil: phenylpropanoids: methylchavicol (58-88 ppm), methyleugenol (0.2%-6%).	-
Origanum vulgare L.	Lamiaceae	fresh leaf	aerial part	Essential oil: bicyclic monoterpene: beta-thujone (0-0.6%), monoterpene etheroxide: 1,8-cineole (0-6.5%).	-
Papaver rhoeas L.	Papaveraceae	fresh leaf	whole plant	Genus in which species may contain isoquinoline alkaloids (morphinanes): e.g. morphine, codeine, rhoeadine.	-
Prunus spinosa L.	Rosaceae	fresh fruit	fruit, leaf and seed	Genus in which species may contain cyanogenic glycosides: e.g. amygdalin, prunasin.	-
Punica granatum L.	Lythraceae	fresh fruit	fruit, root cortex and tree bark	Piperidine alkaloids (0.5-0.7%): e.g. pelletierine, iso-pelletierine, methylisopelletierine and tropane alkaloids: e.g. pseudopelletierine.	The fruit hydroalcoholic extract is genotoxic in vitro and in vivo.
Rosmarinus officinalis L.	Lamiaceae	fresh leaf	aerial part	Essential oil from the herb: bicyclic monoterpenes: e.g. camphor and monoterpene etheroxide: 1,8-cineole (13 to 31%) Essential oil from the leaf: monoterpene etheroxide: 1,8-cineole (11.2-47%) and bicyclic monoterpenes: e.g. camphor (13-31%) and monocyclic monoterpene ketone: pulegone (0.98%).	-
Rubus idaeus L.	Rosaceae	fresh fruit	leaf	-	Oral administration to rats since the start of the gestation until parturation showed an increase of the gestation length. Female offspring (F1) showed precocious puberty age and a significant proportion of their offspring (F2) were growth restricted.
Rumex acetosa L.	Polygonaceae	fresh leaf	whole plant	Genus in which species may contain hydroxyanthracene derivatives and oxalates.	-
Rumex acetosella L.	Polygonaceae	fresh leaf	whole plant	Genus in which species may contain hydroxyanthracene derivatives and oxalates.	-
Rumex scutatus L.	Polygonaceae	fresh leaf	whole plant	Genus in which species may contain hydroxyanthracene derivatives and oxalates.	-
Salvia officinalis L.	Lamiaceae	fresh leaf	aerial part	Essential oil from leaf: bicyclic monoterpenes: e.g.alpha-thujone (12-65%), beta-thujone (1.2-35.6%) (total thujone content 30-60%), camphor (4.4-30%) and monoterpene etheroxide: 1,8-cineole (8-22.5%); phenylpropanoids: e.g. methylchavicol.	-
Sambucus nigra L.	Adoxaceae	fresh fruit	whole plant	Cyanogenic glycoside: S-sambunigrin (3 to 17 mg HCN /100 g fresh weight in leaf and 3 mg HCN / 100g of fruit).	Presence of lectins in branches. Branches, unripe berries or seed of a number of different

					Sambucus species are characterised by the presence of substances able to induce gastrointestinal disorders.
Satureja montana L.	Lamiaceae	fresh leaf	aerial part	Essential oil: monoterpene etheroxide: 1,8-cineole (0.59%) and bicyclic monoterpenes: e.g. camphor (0.21%) and phenylpropanoids: e.g. methyleugenol (25 -415 ppm).	-
Teucrium polium L.	Lamiaceae	fresh leaf	aerial part	Genus in which species may contain furanoneoclerodane diterpenoids: e.g. teucrins.	-

References

1. Sánchez-Mata, M. de C.; Tardío, J. *Mediterranean Wild Edible Plants: Ethnobotany and Food Composition Tables*; Springer New York, 2016; ISBN 9781493933297.
2. Hanen, N.; Fattouch, S.; Ammar, E.; Neffati, M. Allium Species, Ancient Health Food for the Future? *Sci. Heal. Soc. Asp. Food Ind.* **2012**, doi:10.5772/30924.
3. Fang, J. Nutritional Composition of Saskatoon Berries: A Review. *https://doi.org/10.1139/cjb-2019-0191* **2020**, *99*, 175–184, doi:10.1139/CJB-2019-0191.
4. U.S. Department of Agriculture, Agricultural Research Service. FoodData Central, 2019. fdc.nal.usda.gov
5. Viano, J.; Masotti, V.; Gaydou, E.M.; Giraud, M.; Bourreil, P.J.L.; Ghiglione, C. Composition of Liliifloreae from Mediterranean Pastures. *J. Agric. Food Chem.* **1996**, *44*, 3126–3129, doi:10.1021/JF960111A.
6. Iqbal, S.; Younas, U.; Chan, K.W.; Zia-Ul-Haq, M.; Ismail, M. Chemical Composition of *Artemisia annua* L. Leaf and Antioxidant Potential of Extracts as a Function of Extraction Solvents. *Molecules* **2012**, *17*, 6020, doi:10.3390/MOLECULES17056020.
7. Al-Masri, M.R. Nutritive Evaluation of Some Native Range Plants and Their Nutritional and Anti-Nutritional Components. *http://www.tandfonline.com/action/journalInformation?show=aimsScope&journalCode=taar20#.VsXoziCLRhE* **2013**, *41*, 427–431, doi:10.1080/09712119.2013.792733.
8. Silva, C.F.L.; Schirmer, M.A.; Maeda, R.N.; Barcelos, C.A.; Pereira, N. Potential of Giant Reed (*Arundo donax* L.) for Second Generation Ethanol Production. *Electron. J. Biotechnol.* **2015**, *18*, 10–15, doi:10.1016/J.EJBT.2014.11.002.
9. Couplan, F. Guide Nutritionnel des Plantes Sauvages et Cultivées. **2011**.
10. Barreira, J.C.M.; Casal, S.; Ferreira, I.C.F.R.; Oliveira, M.B.P.P.; Pereira, J.A. Nutritional, Fatty Acid and Triacylglycerol Profiles of *Castanea sativa* Mill. Cultivars: A Compositional and Chemometric Approach. *J. Agric. Food Chem.* **2009**, *57*, 2836–2842, doi:10.1021/JF803754U.
11. Ota, A.; Višnjevec, A.M.; Vidrih, R.; Prgomet, Ž.; Nečemer, M.; Hribar, J.; Cimerman, N.G.; Možina, S.S.; Bučar-Miklavčič, M.; Ulrih, N.P. Nutritional, Antioxidative, and Antimicrobial Analysis of the Mediterranean Hackberry (*Celtis australis* L.). *Food Sci. Nutr.* **2017**, *5*, 160–170, doi:10.1002/FSN3.375.
12. Bouhafsoun, A.; Boukeloua, A.; Yener, I.; Lamine, M. Chemical composition and mineral contents of leaflets, rachis and fruit of *Chamaerops humilis* L. *ResearchGate* **2020**.
13. S., B.; P., K.; M., K.; H., K.S. Coriander (*Coriandrum sativum* L.): Processing, Nutritional and Functional Aspects. *African J. Plant Sci.* **2014**, *8*, 25–33, doi:10.5897/AJPS2013.1118.
14. Farrán, A.; Zamora, R.; Cervera, P. *Tablas de Composición de Alimentos del CESNID*; U. de Barcelona, Ed.; 2013.
15. Pedreiro, S.; da Ressurreição, S.; Lopes, M.; Cruz, M.T.; Batista, T.; Figueirinha, A.; Ramos, F. *Crepis vesicaria* L. subsp. *taraxacifolia vesicaria* Leaf: Nutritional Profile, Phenolic Composition and Biological Properties. *Int. J. Environ. Res. Public Heal. 2021, Vol. 18, Page 151* **2020**, *18*, 151, doi:10.3390/IJERPH18010151.
16. Silva, A.R.; Fernandes, Â.; García, P.A.; Barros, L.; Ferreira, I.C.F.R. *Cytinus hypocistis* (L.) subsp. *macranthus* Wettst.: Nutritional Characterization. *Mol. 2019, Vol. 24, Page 1111* **2019**, *24*, 1111, doi:10.3390/MOLECULES24061111.
17. Khan, M.N.; Sarwar, A.; Adeel, M.; Wahab, M.F. Nutritional Evaluation of *Ficus carica* Indigenous to Pakistan. *African J. Food, Agric. Nutr. Dev.* **2011**, *11*, doi:10.4314/AJFAND.V11I5.70445.
18. Khantouche, L.; Guesmi, F.; Motri, S.; Abderabb, M. Nutritional Composition, Analysis of Secondary Metabolites and Antioxidative Effects of the Leaf of *Globularia alypum* L. *Indian J. Pharm. Sci.* **2018**, *80*, 274–281, doi:10.4172/PHARMACEUTICAL-SCIENCES.1000355.
19. Raja, G.; Shaker, I.A.; Inampudi, M. Nutritional Analysis of Nuts Extract of *Juglans regia* L. *Int. J. Bioassays* **2012**, *1*, 68, doi:10.21746/IJBIO.2012.10.001.
20. Maurizi, A.; Michele, A. De; Ranfa, A.; Ricci, A.; Roscini, V.; Coli, R.; Bodesmo, M.; Burini, G. Bioactive Compounds and Antioxidant Characterization of Three Edible Wild Plants Traditionally Consumed in the Umbria Region (Central Italy): *Bunias erucago* L. (Corn Rocket), *Lactuca perennis* L. (Mountain Lettuce) and *Papaver rhoeas* L. (Poppy). *J. Appl. Bot. Food Qual.* **2015**, *88*, 109–114, doi:10.5073/JABFQ.2015.088.015.
21. Aziz, E.E.; Rezk, A.I.; Omer, E.A.; Nofal, O.A.; Salama, Z.A.; Fouad, H.; Fouad, R. Chemical Composition of *Mentha pulegium* L. (Pennyroyal) Plant as Influenced by Foliar Application of Different Sources of Zinc. *Egypt. Pharm. J.* **2019**, *18*, 53, doi:10.4103/EPJ.EPJ_38_18.
22. Andreu, V., Amiot, A., Safont, M., Levert, A., Bertrand, D. First Phytochemical Characterization and Essential Oil Analysis of the Traditional Catalan Wild Salad: "Coscoll" (*Molopospermum peloponnesiacum* (L.) Koch). *Med. Aromat. Plants* **2015**, *04*, doi:10.4172/2167-0412.1000211.
23. Isaac, A.A. Overview of Cactus (*Opuntia ficus-indica* (l): A Myriad of Alternatives. *Stud. Ethno-Medicine* **2016**, *10*, 195–205, doi:10.1080/09735070.2016.11905488.

24. Uddin, M.K.; Juraimi, A.S.; Hossain, M.S.; Nahar, M.A.U.; Ali, M.E.; Rahman, M.M. Purslane Weed (*Portulaca oleracea*): A Prospective Plant Source of Nutrition, Omega-3 Fatty Acid, and Antioxidant Attributes. *Sci. World J.* **2014**, *2014*, doi:10.1155/2014/951019.
25. European Food Safety Authority; Compendium of botanicals reported to contain naturally occuring substances of possible concern for human health when used in food and food supplements. EFSA Journal 2012;10(5):2663. [60 pp.] doi:10.2903/j.efsa.2012.2663. Available online: www.efsa.europa.eu/efsajournalSpecies Database: Biodiversity for Food and Nutrition.
26. Darwish, A.; Hamad, G.; Sohaimy, S. Nutrients and Constituents Relevant to Antioxidant, Antimicrobial and Anti-Breast Cancer Properties of *Salvia officinalis* L. *Int. J. Biochem. Res. Rev.* **2018**, *23*, 1–13, doi:10.9734/IJBCRR/2018/43273.
27. Viano, J.; Masotti, V.; Gaydou, E.M. Nutritional Value of Mediterranean Sheep's Burnet (*Sanguisorba minor* ssp. *muricata*). *J. Agric. Food Chem.* **1999**, *47*, 4645–4648, doi:10.1021/JF981265O.
28. Erden, Y.; Kırbağ, S.; Yılmaz, Ö. Phytochemical Composition and Antioxidant Activity of Some *Scorzonera* Species. *Proc. Natl. Acad. Sci. India Sect. B - Biol. Sci.* **2013**, *83*, 271–276, doi:10.1007/S40011-012-0129-7/TABLES/5.
29. Alghazeer, R.; Elgahmasi, S.; Abdullah, E.; Ahtiwesh, O.; Althaluti, E.; Shamlan, G.; Alansari, W.S.; Eskandrani, A.A. Elucidation of Nutritional, Phytochemical and Pharmacological Activities of *Teucrium pol*ium l Grown in Libya. *J. Anim. Plant Sci.* **2021**, *31*, 1439–1452, doi:10.36899/JAPS.2021.5.0345.
30. Petropoulos, S.A.; Fernandes, Â.; Tzortzakis, N.; Sokovic, M.; Ciric, A.; Barros, L.; Ferreira, I.C.F.R. Bioactive Compounds Content and Antimicrobial Activities of Wild Edible Asteraceae Species of the Mediterranean Flora under Commercial Cultivation Conditions. *Food Res. Int.* **2019**, *119*, 859–868, doi:10.1016/J.FOODRES.2018.10.069.
31. HMPC Assessment Report on *Urtica dioica* L., and *Urtica urens* L., herba. **2008**.
32. Ercisli, S. Chemical Composition of Fruit in Some Rose (*Rosa* spp.) Species. *Food Chem.* **2007**, *104*, 1379–1384, doi:10.1016/J.FOODCHEM.2007.01.053.
